# Socioeconomic Disparities in Breast Cancer Screening in Hawaii

**Published:** 2007-09-15

**Authors:** Timothy Halliday, Deborah A Taira, James Davis, Henry Chan

**Affiliations:** John A. Burns School of Medicine; University of Hawaii at Manoa; Hawaii Medical Service Association (an independent licensee of the Blue Cross and Blue Shield Association) and John A. Burns School of Medicine, Honolulu, Hawaii; Hawaii Medical Service Association (an independent licensee of the Blue Cross and Blue Shield Association) and John A. Burns School of Medicine, Honolulu, Hawaii; Hawaii Medical Service Association, Honolulu, Hawaii

## Abstract

**Introduction:**

Despite evidence that breast cancer screening reduces morbidity and mortality, many women do not obtain mammograms. Our objective was to analyze the relationship between income and mammography screening for members enrolled in a large health plan in Hawaii.

**Methods:**

We analyzed claims data for women (N = 46,328) aged 50 to 70 years during 2003 and 2004. We used parametric and nonparametric regression techniques. We used probit estimation to conduct multivariate analysis.

**Results:**

At the 5th percentile of the earnings distribution, the probability of mammography is 57.1%, and at the 95th percentile, it is 67.7%. Movement from the 5th percentile to the 35th percentile of the earnings distribution increases the probability of mammography by 0.0378 percentage points. A similar movement from the 65th percentile to the 95th percentile increases the probability by 0.0394 percentage points. Also, we observed an income gradient within narrowly defined geographic regions where physical access to medical care providers is not an issue.

**Conclusion:**

We observed a steep income gradient in mammography screening in Hawaii. Because of the prevalence of measurement error, this gradient is probably far greater than our estimate. We cannot plausibly attribute our findings to disparities in coverage because 100% of our sample had health insurance coverage. The gradient also does not appear to result from poorer people residing in areas that are geographically isolated from providers of medical care.

## Introduction

According to the U.S. Preventive Services Task Force, mammography screening is estimated to reduce mortality from breast cancer by 20% to 30% (1). Despite this reduction in mortality, however, mammography is greatly underused by women who are at risk for breast cancer (2-5). Moreover, the problem of underutilization of mammography is exacerbated by low socioeconomic status (SES). Studies show that use is low among African Americans (6), people with low levels of education (7,8), and people with low incomes (7). Policies designed to increase the use of preventive medicine among the poor may play a positive role in mitigating the gradient or the ubiquitous correlation between SES and health (9,10).

In this study, we investigate the relationship between race and income and breast cancer screening in Hawaii. The data used are well suited for this investigation for two reasons. First, we used claims data from a large health plan, not patient self-reports, which tend to overstate mammography use (2). Second, we also used U.S. census income data by census tract, whereas other studies have used wide income brackets (11) or census data by zip code (3).

## Methods

### Sample selection and variable descriptions 

We analyzed claims data from a large health insurance plan in Hawaii that provides coverage to approximately half of the state's population. In our study, we included women (N = 46,328) aged 50 to 70 years during 2003 and 2004. The sample contained variables on breast cancer screening, age, insurance plan type, morbidity level, income, and race. Table 1 provides variable definitions and descriptive statistics for our sample. Our outcome of interest was a variable that indicates whether women in our sample had a mammogram during the years 2003 or 2004.

We used income data from the 2000 U.S. census. For each census tract, we computed median income using two measures: family income and per capita family income, which is family income divided by the number of people in the household. We then used global positioning system software to determine the census tract of each member in our sample and merged the income data by census tract into the claims database using member addresses. Our data covered a total of 280 census tracts.

We obtained data on race from a member satisfaction questionnaire administered by the health plan each year to a random sample of its members. We obtained self-reported race information for 38% of the study population (Table 1). However, because nonresponse may not have been random, this 38% probably does not constitute a random sample. To address this issue, we constructed a dummy variable indicating whether or not the race data were missing.

We used a morbidity index from the Adjusted Clinical Group case-mix adjustment system, which categorizes a patient's clinical conditions from the *International Classification of Disease, Ninth Revision* into one of six integer categories ranging from zero through five (12). Higher numbers indicate worse morbidity. This measure of morbidity is a risk-adjustment tool that measures the illness burden of patients and their expected consumption of health services. We calculated the median value of the index for 2003 and 2004 (Table 1).

### Breast cancer screening  

To identify members who had breast cancer screening, we used the following codes from the Health Plan Employer Data and Information Set (HEDIS), which is used by more than 90% of America's health plans to measure performance on dimensions of care and service (13):

- Primary Diagnosis Code: V76.11, V76.12 or
- Additional Diagnosis Code: V76.11, V76.12 or
- Surgical Procedure Code: 87.36, 87.37

### Regression techniques

We used two regression techniques. First, we estimated nonparametric local linear regressions (14), which allowed us to see how the propensity to obtain mammography screening responds to income without imposing any parametric assumptions on the data (15). Although nonparametric regression enabled us to investigate the relationship between mammography and income as flexibly as possible, it was a cumbersome task to conduct a multivariate analysis with nonparametric techniques because of data limitations and what is known in statistics and econometrics as the "curse of dimensionality." Thus, we turned to probit estimation to conduct our multivariate analysis. Multivariate probit models allowed us to quantify the impact of SES on the probability of mammography screening while controlling for confounding factors. We calculated the marginal effects for each of the variables in our probit regressions. The marginal effect for the *i*th covariate is defined as 

$\frac{\partial P(y|x)}{\partial x_{i}}=ø(\overset{-}{x}\beta )\beta_{i}$

where *ø*(.) is the probability density function of a standard normal random variable, *x* is the mean of our data vector, and *β*_*i*_ is the coefficient on the *i*th covariate (16). To address concerns about correlations among observations within census tracts, we adjusted the standard errors of our coefficient estimates for clustering within census tracts (15). We used Stata (StataCorp LP, College Station, Texas) for all estimation procedures.

## Results

### Breast cancer screening rates

The overall screening rate for women in our sample was 64% for the 2-year study period. Figure 1 shows mammography rates by age group in Hawaii. We found no differences in screening rates among age groups.

**Figure:**
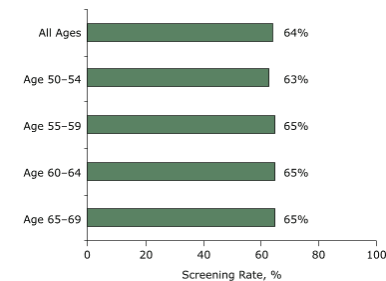
Breast cancer screening rates by age group, Hawaii, 2003–2004.

### Regression results

Figure 2 displays the results obtained by estimating a local-linear regression of the mammography dummy on the log of per capita household income. In the figure, we predict the probability of mammography for incomes ranging from the bottom 5th percentile to the top 95th percentile of the earnings distribution. The figure shows a wide disparity in the demand for mammography screening by income. At the lowest end, the probability of obtaining a mammogram is 57.1%, whereas at the highest end, the probability is 67.7%.

**Figure:**
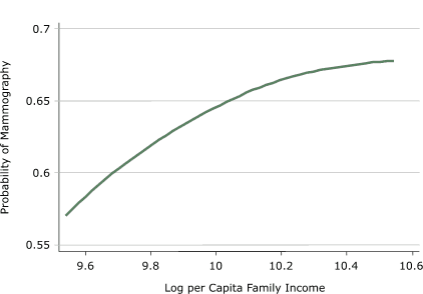
Probability of obtaining a mammogram among a sample of women aged 50 to 70 years, for incomes ranging from the 5th percentile to the 95th percentile of the earnings distribution, Hawaii, 2003–2004.

Table 2 presents the results of probit models. In columns 1 and 2 of Table 2, we report the correlation between per capita family income and the probability of obtaining a mammogram without any additional control variables (column 1) and with a set of age dummies (column 2). Both estimates are positive and significant. A 1% increase in per capita family income is associated with an increase in the probability of obtaining a mammogram of 0.001 percentage points. In less infinitesimal terms, calculations based on the estimates in column 2 suggest that movement from the 5th percentile to the 35th percentile of the earnings distribution increases the probability of mammography by 0.0378 percentage points. A similar movement from the 65th percentile to the 95th percentile has an effect of 0.0394 percentage points. As in Figure 1, we do not see any substantial variation in mammography screening among age groups.

Column 3 is identical to column 2 except that we use family income in lieu of per capita family income. We now see that the marginal effect of income is cut by roughly 22.5%. Presumably, the reason for this decrease is that per capita income is a better proxy for the family's living standards. Thus, we expect less attenuation bias when using per capita family income than when using total family income.

In columns 4 and 5, we control for the member's morbidity level. Column 4 includes the actual morbidity index, and column 5 includes dummy variables for each morbidity level. We see that the higher morbidity levels as measured are highly correlated with obtaining a mammogram. We also see that the inclusion of morbidity controls does not alter the estimated effect of income.

In column 6 of the table, we include dummy variables indicating the member's location. The locations are east Hawaii, west Hawaii, Kauai, Lanai, Maui, Molokai, Honolulu, and Oahu other than Honolulu. We see that inclusion of these dummies increases the effect of income by 28.6%. The inclusion of regional dummies identifies the relationship between changes in income and mammography screening within regions of Hawaii. Moreover, the higher coefficient on income associated with the regional dummies suggests a relationship between income and mammography screening within regions of Hawaii.

In column 7, we add a dummy variable indicating whether or not the individual is a member of a health maintenance organization (HMO). The HMO dummy is positive and significant. While holding other factors constant, we see that belonging to an HMO increases the probability of mammography screening by 0.016, which constitutes a 2.5% increase in the mean probability of obtaining a mammogram.

Column 7 also includes a set of race dummies. We see some variation across race. Mammography screening is most common among Chinese women, who are followed by Japanese women. The dummy variable indicating missing race data is negative and significant, suggesting that race data are not missing randomly.

Table 3 provides more details for column 5 of Table 2. We show results only for east Hawaii, Maui, Honolulu, and parts of Oahu other than Honolulu because other regions of the state yielded samples that were too small. Although some regions such as east Hawaii and Maui have reasonable sample sizes, they lack a large number of census tracts, which tend to be concentrated on Oahu. Accordingly, we may still expect imprecise estimates for these regions. Table 3 shows that even within narrowly defined geographic regions, the demand for mammography by income varies, consistent with column 6 of Table 2.

## Discussion

### Main findings

The overall screening rate of 64% in our sample is broadly consistent with other estimates of mammography (2,3). It is important to emphasize, however, that estimates using self-reported data tend to be higher than estimates using insurance or hospital records (2,4).

We document a large disparity in mammography use across the earnings distribution in Hawaii. At the 5th percentile of the earnings distribution, the probability of mammography is 57.1%, and at the 95th percentile, it is 67.7%. We find that a 1% increase in income increases the probability of having a mammogram by 0.001. We emphasize that our measures of family income contain error because they measure only median income in a given census tract. Given the conventional wisdom that classical measurement error will tend to attenuate coefficient estimates, it is reasonable to expect that the true relationship between income and mammography screening is far greater than we have estimated (17). In other words, the real problem is probably far worse than we document.

We estimate a stronger relationship between income and mammography screening than other studies that use multivariate probit analysis (3,11). There are two reasons for the stronger relationship. First, we merged in income by census tract, whereas other studies have used income by zip code (which is coarser) or have used wide income brackets. The second reason for the stronger relationship is that we used income per household member. For both of these reasons, we have a more precise measure of family income, which mitigates the attenuation bias that results from less well-measured income.

The large disparity in mammography across the earnings distribution observed in our study is interesting for two reasons. First, despite having 100% coverage of mammography in our sample, we still see a higher demand for preventive medical care among the rich than among the poor. Income plays a large role in a population where everybody has health insurance and there are no out-of-pocket expenses for obtaining mammograms. While universal health coverage may mitigate socioeconomic disparities in the demand for preventive medicine, as suggested by the Rand Health Insurance Experiment (18), our findings suggest that universal health coverage will not eliminate disparities. Rather, our findings suggest that a gradient in the consumption of prophylactic health care would persist even with universal coverage. Second, we saw that the income gradient exists within narrowly defined geographic regions such as the County of Honolulu, where physical access to medical care providers is not an issue. The observed socioeconomic gradient in the demand for mammography screening does not appear to be the consequence of poorer people residing in areas that are geographically isolated from providers of medical care.

We also determined that higher morbidity levels are highly correlated with obtaining a mammogram. To understand the positive coefficient on the morbidity index in Table 2, it is important to note that many measures of morbidity tend to be highly correlated with the patient's use of medical services or medical demand (19). Moreover, many studies have also shown that physician recommendation, which is more likely to occur when use of medical services is high, is a strong predictor of mammography screening (3,5,20-22). Given this finding, it is not surprising that the morbidity index is such a strong predictor of mammography screening.

The effect of income was not altered by the inclusion of morbidity controls in Table 2, which is curious, given that a strong correlation between income and health has been documented in virtually every context imaginable (9,10). This result is less surprising, however, when one considers that our morbidity index is probably measuring medical demand, at least to some extent, and that medical demand is a function of both income and health status. Each function tends to affect the demand for medical care in opposing ways. On one hand, because richer people tend to consume more medical services, the inclusion of the morbidity controls should attenuate the effect of income, holding all other factors equal. On the other hand, poorer people tend to be sicker people who, other factors held constant, consume more medical services, thus causing the inclusion of morbidity controls to increase the effect of income. The existence of these two countervailing effects suggests that the inclusion of the morbidity controls would have no net impact on the estimated effect of income.

Belonging to an HMO increased the probability of mammography screening in our study. Differing pecuniary incentives do not explain this finding because there is no cost to the individual for mammography in either the preferred provider organization (PPO) plan or the HMO plan. However, HMO members of the health care plan in this study are required to choose a principal provider for their care and, as part of the health plan's quality care initiative, principal providers receive lists of patients who do not receive mammograms. This practice is not part of the PPO plan, a difference that is consistent with the commonly held notion that HMOs tend to place a greater emphasis on preventive care than do PPOs. Nevertheless, other studies do not find any relationship between mammography and HMO participation (11).

Finally, our suggestion that race data are not missing randomly (Table 2, column 6) provides an important caveat to researchers who wish to use voluntary questionnaires to make inferences about population relationships.

So, if income gradients in mammography use are not caused by lack of coverage or geographic isolation, then what is responsible for them? We explore some possible explanations below.

### Mechanisms 

#### Ex ante moral hazard

One possible explanation for our findings is ex ante moral hazard, or the notion that insurance coverage for curative care reduces the incentive for investing in preventive care (23,24). The issue of ex ante moral hazard does not explain our results for several reasons. First, for ex ante moral hazard to be responsible for our results, coverage of curative care would have to differ systematically between rich and poor members, which it does not. Second, although insurance at least partially mitigates the costs of cancer treatment, risks such as increased probability of death due to late detection remain even with comprehensive insurance coverage. Given this information, it is not surprising that evidence of ex ante moral hazard is scant in the literature (23).

#### Time costs

Another possible explanation for our results is that poorer people incur a higher time cost for obtaining a mammogram. For example, richer people may have more flexible employment, enabling them to take time off work with little or no effect on their earnings. Poorer people may also tend to have wage-based rather than salaried jobs, meaning that they must forgo valuable work time to see a physician or obtain a mammogram. Moreover, poorer people may rely more heavily on public transportation than do richer people, which would also tend to increase the time cost of obtaining medical care. Indeed, evidence exists that these time costs may be particularly important among Asian Americans (25), of whom there are many in Hawaii. Thus, time costs may be an important mechanism in this study.

#### Information

A third possible explanation for our results is that poorer members are less informed than richer members about the potential benefits of mammography screening. Although the benefits of early detection are well-documented in the literature, this information may not be disseminated equally across the earnings distribution. Indeed, some evidence suggests that race, which is highly correlated with income, is a significant predictor of attitudes toward the efficacy of screening for breast and cervical cancer (26,27).

### Limitations and further work 

Our study has several limitations. First, data are from a single health plan in Hawaii and may not generalize to other settings or populations. Second, we had race information available only on a subset of members who had seen their doctor in the past year. Racial disparities in breast cancer screening for the general population may differ from our results. Third, information on relevant factors such as health beliefs, transportation, and family history of disease was not available. Fourth, we used median income level by census tract rather than an individual's actual income, which introduces measurement error.

Despite these limitations, our findings suggest that an income gradient exists in the probability of obtaining breast cancer screening, with low-income women being less likely than high-income women to receive screenings. To address this disparity, further research will be needed to identify the reasons for lack of compliance with recommended guidelines. For instance, we could use chart data to determine how often physicians schedule screenings that patients fail to attend. Analyzing barriers to breast cancer screenings from the patient perspective is also of interest.

## Figures and Tables

**Table 1:** Sample (N = 46,328) Characteristics, Study on Socioeconomic Disparities in Breast Cancer Screening in Hawaii, 2003–2004

Characteristic	Value
**Age, y, mean (SD)**	58.4 (5.4)
**Health plan type, % of individuals**	
Health maintenance organization	20
Preferred provider organization	80
**Morbidity level, % of individuals^a^**	
0	2.1
0.5	2.7
1	6.1
1.5	9.0
2	14.1
2.5	17.6
3	21.3
3.5	10.7
4	8.3
4.5	4.3
5	3.7
**Median annual income in member's census tract, mean (SD), 2000 dollars^b^**	
Per family income	65,024 (19,114)
Per capita family income	24,132 (7,884)
**Race reported, % of individuals^c^**	38
Chinese	7
Japanese	40
Filipino	9
Korean	2
Hawaiian	11
White	18
Mixed race	8
Other race	4
**Race not reported, % of individuals**	62

a We used a morbidity index from the Adjusted Clinical Group case-mix adjustment system, which categorizes a patient's clinical conditions into one of six integer categories ranging from zero through five (12). Higher numbers indicate worse morbidity. We used the median value of the index for 2003 and 2004.

b Sample size is 46,320.

c These values correspond only to the subsample of members who responded to the member satisfaction questionnaire.

**Table 2:** Probit Models in Which the Dependent Variable is an Indicator for Having Had a Mammogram, Study on Socioeconomic Disparities in Breast Cancer Screening in Hawaii, 2003–2004^a^

Category	Variables						

	Per Capita Family Income		Family Income	Morbidity Level		Region	Race and Member of Health Maintenance Organization (HMO)

	No Additional Controls	Dummy Variables for Age		Dummy Variables for Age			

				Actual Morbidity Index	Dummy Variables for Morbidity Category	Dummy Variables for Morbidity Levels	

						Dummy Variables for Member Location	Dummy Variable for Member Location

							Dummy Variables for Race and for HMO Member or Not
Column no.	1	2	3	4	5	6	7
No. in sample	46,328	46,328	46,320	46,328	46,328	46,328	46,328
**Income**							
Log per capita family income	0.098 (7.30)	0.098 (7.27)	—	0.099 (7.31)	0.098 (7.18)	0.126 (10.06)	0.110 (9.72)
Log family income	—	—	0.076 (6.09)	—	—	—	—
**Age, y**							
Ages 50 to 54	—	−0.017 (−2.21)	−0.021 (−3.33)	0.026 (3.36)	0.016 (2.00)	0.014 (1.76)	0.021 (2.67)
Ages 55 to 59	—	−0.003 (−0.41)	−0.007 (−1.10)	0.026 (3.58)	0.018 (2.48)	0.017 (2.39)	0.022 (3.07)
Ages 60 to 64	—	0.005 (0.38)	−0.005 (−0.73)	0.018 (2.42)	0.011 (1.46)	0.010 (1.39)	0.014 (1.79)
**Member of HMO**	—	—	—	—	—	—	0.016 (2.67)
**Race**							
Chinese	—	—	—	—	—	—	0.082 (3.46)
Filipino	—	—	—	—	—	—	−0.015 (−0.60)
Japanese	—	—	—	—	—	—	0.060 (2.91)
Korean	—	—	—	—	—	—	0.058 (1.66)
Hawaiian	—	—	—	—	—	—	−0.014 (−0.61)
White	—	—	—	—	—	—	0.030 (1.37)
Mixed race	—	—	—	—	—	—	0.002 (0.01)
Race data missing	—	—	—	—	—	—	−0.063 (−3.17)
**Morbidity level**	—	—	—	0.086 (38.64)	—	—	—
**Pseudo R-squared**	0.0031	0.0033	0.0020	0.0327	0.0569	0.0609	0.0688

Dashes (—) indicate that data do not apply.

a The top number in each cell, unless otherwise indicated, is the marginal impact of the corresponding variable and the bottom number, in parentheses, is the *t *statistic corresponding to the underlying coefficient. All standard errors adjust for clustering within census tracts.

**Table 3:** Income Gradients by Region, Study on Socioeconomic Disparities in Breast Cancer Screening in Hawaii, 2003–2004^a^

Region	Marginal Effect of Log per Capita Family Income		

	Marginal Impact of Income	*t* Statistic	Sample Size
East Hawaii	0.18	3.60	2,953
Maui	0.07	1.41	3,036
Honolulu	0.12	(6.35)	19,173
Oahu (other than Honolulu)	0.15	(9.40)	18,752

a This table contains the results of probit models in which the dependent variable is an indicator for having had a mammogram within the past 2 years. Each regression contains age and morbidity dummies. All standard errors adjust for clustering within census tracts.
